# Chemical Reactivity of Strongly Interacting, Hydrogen-Bond-Forming Molecules Following 193 nm Photon Irradiation: Methanol in Amorphous Solid Water at Low Temperatures

**DOI:** 10.1021/acs.langmuir.2c03441

**Published:** 2023-02-10

**Authors:** Michelle Sykes Akerman, Hiley Iny, Roey Sagi, Micha Asscher

**Affiliations:** Institute of Chemistry, Edmund J. Safra Campus, Givat Ram, The Hebrew University of Jerusalem, Jerusalem 91904, Israel

## Abstract

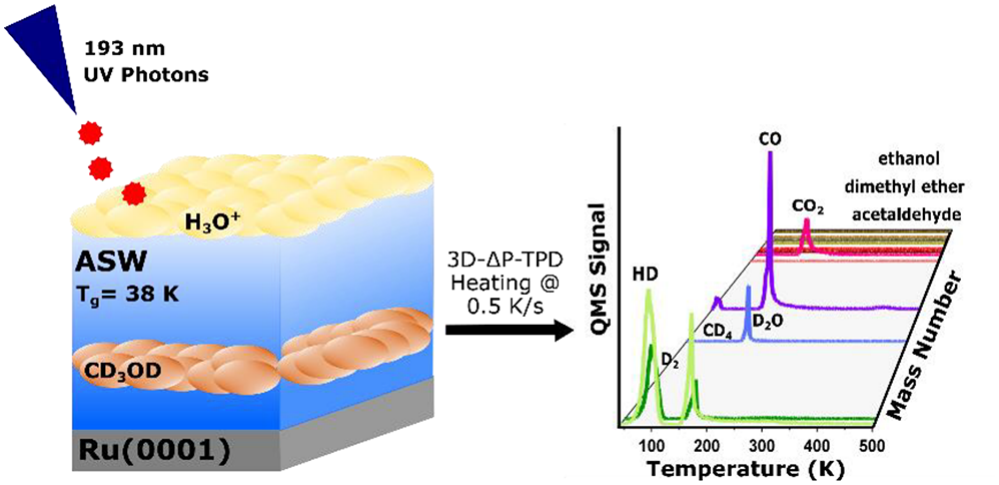

Mixtures of methanol and amorphous solid water (ASW) ices are observed in the interstellar medium (ISM), where they are subject to irradiation by UV photons and bombardment by charged particles. The charged particles, if at high enough density, induce a local electric field in the ice film that potentially affects the photochemistry of these ices. When CD_3_OD@ASW ices grown at 38 K on a Ru(0001) substrate are irradiated by 193 nm (6.4 eV) photons, products such as HD, D_2_, CO, and CO_2_ are formed in large abundances relative to the initial amount of CD_3_OD. Other molecules such as D_2_O, CD_4_, acetaldehyde, and ethanol and/or dimethyl ether are also observed, but in smaller relative abundances. The reactivity cross sections range from (2.6 ± 0.3) × 10^–21^ to (3.8 ± 0.3) × 10^–25^ cm^2^/photon. The main products are formed through two competing mechanisms: direct photodissociation of methanol and water and dissociative electron attachment (DEA) by photoelectrons ejected from the Ru(0001) substrate. An electric field of 2 × 10^8^ V/m generated within the ASW film during Ne^+^ ions bombardment is apparently not strong enough to affect the relative abundances (selectivity) of the photochemical products observed in this study.

## 1 Introduction

Water ices are known to be versatile templates for numerous applications including the study of nanoparticle growth^1^ and reactivity^2−5^ as well as photochemistry.^6−9^ Ice grains are used as platforms to study the photodegradation of organic pollutants in cold environments on earth.^8,9^ Amorphous solid water (ASW) films, whose growth can be characterized via their spontaneous polarization as detected by contact potential difference measurements,^10^ are used to study photochemical reactions of astrochemical relevance.^6,7^

The study of the photoprocessing of mixed ices of ASW and methanol is relevant to the field of astrochemistry. In the interstellar medium (ISM), dust grains are coated with water dominated ices that also contain other small molecules such as CH_3_OH, CO, CO_2_, NH_3_, and CH_4_,^11,12^ in varying abundances with respect to water. Methanol is reported to be the most abundant (nondiatomic) molecule after water, ranging from 5 to 50% relative to water.^11,13−15^ In addition, methanol is an important precursor for the formation of other complex organic molecules, including methyl halides and amino acids.^16−18^ The ice-coated dust grains are subject to bombardment by high-energy ions, electrons, and UV photons, which could lead to various new complex molecules. Processing of these ices by direct UV photon absorption results in the formation of radicals that may recombine to form new molecules. In addition, secondary photoelectrons produced by these energetic processes can contribute to the chemistry observed in the ISM through other mechanisms such as dissociative electron attachment (DEA).

Although it is unlikely to occur under ISM conditions, it is nevertheless interesting to consider the possible effect of charged ice films on the outcome of photochemical processes within such films or nanocapacitors. At low enough temperatures charges become embedded in icy layers, generating a local electric field. This electric field could affect the stability (e.g., orientation) and therefore the reactivity of the radicals^19−22^ and other reactive fragments produced in the water-dominated ices through photon, ion, or electron irradiation. The photochemistry of molecules can also potentially be affected by an electric field induced by a charged functional group.^23^ Generally, strong electric fields are theoretically predicted to stabilize transition states and orient molecules more favorably, enabling otherwise impossible reaction pathways to become permissible.^24−26^

In this study, we aim to explore the outcome of UV irradiation of methanol embedded in ASW at 193 nm (6.4 eV). In addition, we have studied the potential effect of a static electric field on such photochemistry. Water molecules dissociate upon irradiation by UV photons at 193 nm,^27−30^ producing hydrogen atoms and hydroxyl radicals. The photochemistry of amorphous solid methanol at 193 nm has not been extensively investigated. Öberg et al. studied the UV photochemistry of methanol ices using a broad-band UV hydrogen microwave discharge lamp and found that a variety of complex molecules such as ethane, ethanol, dimethyl ether, formaldehyde, and acetaldehyde are formed upon sample heating in addition to smaller molecules such as methane, carbon monoxide, and carbon dioxide.^31^ The photochemistry of methanol at 193 nm has mostly been investigated (theoretically and experimentally) in the gas phase.^32−34^ According to one study, in the solid phase, methanol does not absorb UV light at wavelengths longer than 185 nm.^35^ At shorter wavelengths, such as 157 nm, researchers have shown that the irradiation of amorphous solid methanol leads to the formation of methyl and hydroxyl radicals^36^ as well as hydrogen molecules.^37^ The photochemistry of supported solid mixtures of methanol and ASW at a wavelength of 193 nm has not been reported to the best of our knowledge.

Water–ice nanocapacitors have been used to study the reorientation of molecules,^38,39^ the Stark effect on various molecular vibrations,^40,41^ conformational changes of molecules,^42^ and acid–base proton transfer^43^ under the influence of a strong electric field. So far, this system has not yet been used to study the effect of a strong electric field on the photochemistry of molecules in condensed molecular films. In this study we utilize the ice–nanocapacitor method to study the potential influence of a strong (positive) electric field (∼2 × 10^8^ V/m) on the photochemistry of CD_3_OD@ASW sandwich films at an irradiation wavelength of 193 nm.

## 2 Experimental Section

Sandwich films of deuterated methanol (CD_3_OD) and ASW (H_2_O) are prepared at 38 K on a Ru(0001) single-crystal substrate held at the center of an ultrahigh-vacuum (UHV) chamber with a base pressure of 2 × 10^–10^ Torr. The 8 × 8 × 2 mm^3^ Ru(0001) substrate is spot-welded to two tantalum wires, 0.4 mm in diameter, which are connected to an *x*–*y*–*z* rotatable stage (McAllister) via thick tantalum wires, 3 mm in diameter. The sample can be cooled to a minimum temperature of 30 K using a closed cycle He cryostat (Lakeshore/Janis). A Lakeshore 335 controller is used to stabilize and heat the sample in the temperature range of 30–300 K, utilizing a silicon diode sensor that monitors the temperature at the sample holder. The temperature at the sample is measured to an accuracy of ±1 K through a K-type thermocouple (chromel–alumel) that is spot-welded to one side of the Ru(0001) substrate. A LabView algorithm is used to control the heating and cooling of the sample in the temperature range of 30–1450 K. The sample is cleaned on a daily basis by sputtering with 1000 eV Ne^+^ ions for a duration of 15 min, followed by 10 min annealing at 1450 K. Films of CD_3_OD and H_2_O were prepared by backfilling the chamber with the designated gaseous species until the desired thickness was achieved in units of langmuirs (1 L = 10^–6^ Torr s). For CD_3_OD and H_2_O the conversion from units of langmuirs (L) to monolayers (ML) is determined by identifying the onset of multilayer desorption of each respective species in exposure-dependent temperature-programmed desorption (ΔP-TPD) experiments. For the ΔP-TPD experiments we employ a quadrupole mass spectrometer (QMS, RGA 200, SRS) which detects species by their mass as they desorb from the substrate, while the substrate is heated at a fixed rate (typically 0.5 or 1 K/s). The CD_3_OD@ASW sandwich films are irradiated by UV photons at 193 nm (6.4 eV) generated by an ArF excimer laser (PSX-100). The laser is operated at 50 Hz, with a pulse energy of 1.5 mJ/pulse at the sample (considering the prism and viewport absorbance) with the total number of photons at the sample surface ranging from 1.6 × 10^19^ to 4.7 × 10^20^ photon/cm^2^. The photoproducts are analyzed using a 3D-ΔP-TPD procedure, which scans a mass range of *m*/*z* = 3–16 amu and *m*/*z* = 19–70 amu while the substrate is being heated at a fixed rate of 0.5 K/s. This range excludes the main masses of H_2_O (*m*/*z* = 17, 18) which would lead to saturation of the QMS, obscuring the rest of the ΔP-TPD data. After the 3D-ΔP-TPD data are analyzed and the masses (*m*/*z*) of the main products are identified, the photochemistry experiment is repeated with a regular ΔP-TPD measurement that includes the most abundant masses (*m*/*z*) detected in the 3D run (up to 9 masses simultaneously in a single ΔP-TPD run). Strong (positive) electric fields are generated within the CD_3_OD@ASW sandwich films by charging the films with 85 eV Ne^+^ ions for 10 min to obtain a maximum electric field strength of 2 × 10^8^ V/m, which is stable for more than 20 min.^44^ To determine the effect of the electric field on the photochemistry of CD_3_OD@ASW films, ΔP-TPD experiments were conducted on films that were first irradiated with 193 nm photons at a fixed fluence of 3.1 × 10^20^ photons/cm^2^ and subsequently charged by the 85 eV Ne^+^ ions to obtain a maximum field strength.

## 3 Results and Discussion

In this study we aim to explore the 193 nm photochemistry of methanol (CD_3_OD) in H_2_O-ASW films at low temperatures. Additionally, the possible effect of a strong electric field (2 × 10^8^ V/m) on the photochemistry of these films is investigated. This study was conducted at 38 K to mimic conditions in some areas of the ISM. Additionally, at 38 K the CD_3_OD molecules are not dispersed throughout the ASW films when deposited in sandwich structures.^45^ This means that the position of the CD_3_OD molecules relative to the Ru(0001) substrate can be controlled, and the effects of the substrate on the photochemistry can be explored.

### 3.1 Optimization of the Photochemistry Setup

A total film thickness of 100 ML (including both H_2_O and CD_3_OD) was chosen in order to ensure that the charging process (following Ne^+^ ion collisions^44^) would lead to the formation of a stable positively charged model nanocapacitor. The maximum charge capacity of ASW films and its stability decrease when the films are thinner than 50 ML. This means that the electric fields generated in these films are weaker and decay faster than for films that are thicker than 50 ML. In addition, a film thickness of 100 ML minimizes the recombination between photoelectrons emitted from the Ru(0001) upon 193 nm photon irradiation and the positive charges generated on the ASW film–vacuum interface due to Ne^+^ ion bombardment.

**Figure 1:**
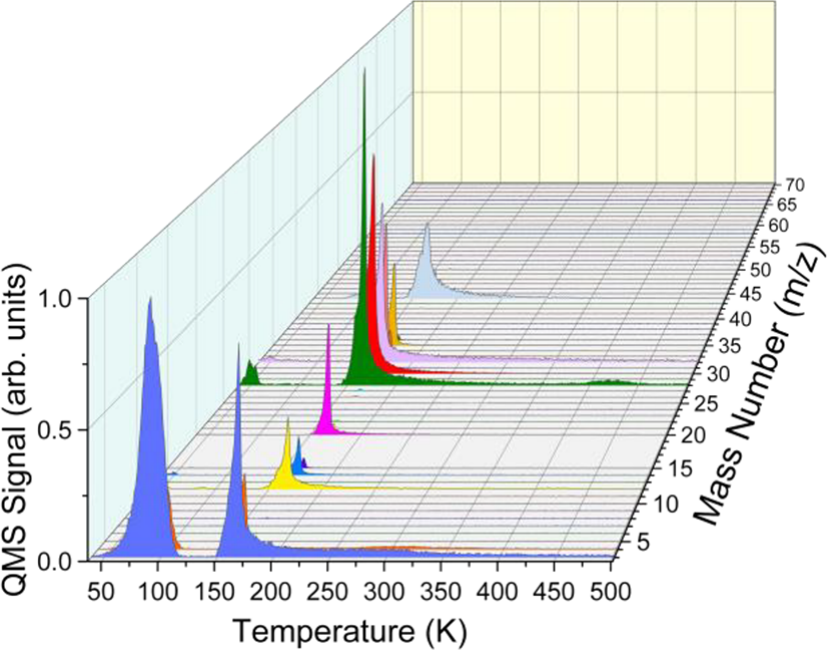
Example of a 3D-ΔP-TPD plot following 193 nm photon irradiation of a 80 ML (H_2_O) ASW/10 ML CD_3_OD/10 ML (H_2_O) ASW/Ru(0001) sandwich film at 38 K.

The amount of CD_3_OD in the ASW films is varied in order to determine the amount of CD_3_OD necessary for producing detectable photochemical products. ASW films containing 5, 10, and 20 ML of CD_3_OD are studied. The CD_3_OD layers are placed on top of 10 ML of H_2_O and covered by H_2_O to bring the film to a total thickness of 100 ML [(90 – *X*) ML (H_2_O)ASW/*X* ML CD_3_OD/10 ML (H_2_O)ASW/Ru(0001)]. The CD_3_OD films are irradiated by 193 nm photons at a fluence of 3.1 × 10^20^ photons/cm^2^. Once the irradiation is complete, 3D-ΔP-TPD experiments are conducted (see Figure 1). In this type of measurement, the QMS signal (related to the pressure of the desorbing species, Δ*P*) is measured for masses (*m*/*z*) in the defined range as the film is heated at a fixed rate. In this way, new masses that were not observed for nonirradiated films can be identified and attributed to photochemical products.

**Figure 2:**
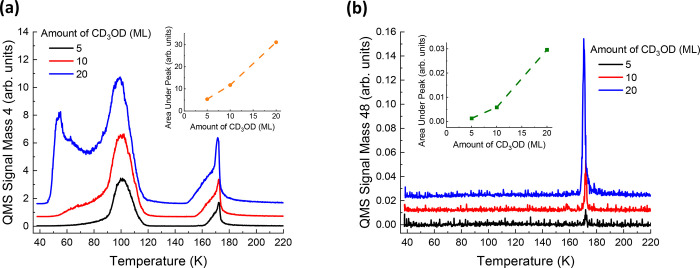
Effect of initial dose of CD_3_OD on the amounts of products produced through 193 nm photon irradiation of sandwich films of [(90 – *X*) ML (H_2_O) ASW/*X* ML CD_3_OD/10 ML (H_2_O) ASW/Ru(0001)] at 38 K. ΔP-TPD profiles for (a) *m*/*z* = 4 and (b) *m*/*z* = 48 show that as the initial amount of CD_3_OD increases, the yield of both *m*/*z* = 4 and *m*/*z* = 48 increases, indicated by increasing area under the ΔP-TPD profile for each indicated mass. The ΔP-TPD profiles are offset for clarity.

Products are detectable for all amounts of CD_3_OD studied here, but the S/N level is low for the higher mass products when there is only 5 ML of CD_3_OD in the ASW film. The yield of the products increases as the initial amount of CD_3_OD in the ASW film increases. However, this increase occurs in a nonlinear pathway, as shown for masses *m*/*z* = 4 and *m*/*z* = 48 (chosen as representative *m*/*z* for small and relatively large products, respectively) in Figures 2a, and 2b. Therefore, we decided to continue all experimentation with 10 ML CD_3_OD in ASW.

The optimal location of the CD_3_OD layers in the ASW films is determined by varying the initial location of the CD_3_OD layers within the ASW film. Three positions are explored: CD_3_OD near the bottom, in the middle, and near the top of the ASW film (near the ASW–vacuum interface). CD_3_OD is not placed directly on the Ru(0001) substrate or on the ASW–vacuum interface in order to maintain a sandwich structure, in which the layers of CD_3_OD molecules are surrounded by H_2_O molecules. The largest amounts of products are observed when CD_3_OD is initially placed near the bottom of the ASW film [80 ML (H_2_O) ASW/10 ML CD_3_OD/10 ML (H_2_O) ASW/Ru(0001)]. In the following sections, the 193 nm photochemistry of films composed of this type of sandwich structure grown and irradiated at 38 K is presented. In Section 3.2, the masses of the products observed in post-photon irradiation ΔP-TPD experiments are presented and assigned to possible products. In Section 3.3, mechanisms of formation of the products are proposed. Finally, in Section 3.4, the potential effect of a strong positive electric field on the photochemistry of these films is discussed.

### 3.2 Photochemistry of Methanol in ASW at 38 K

**Figure 3:**
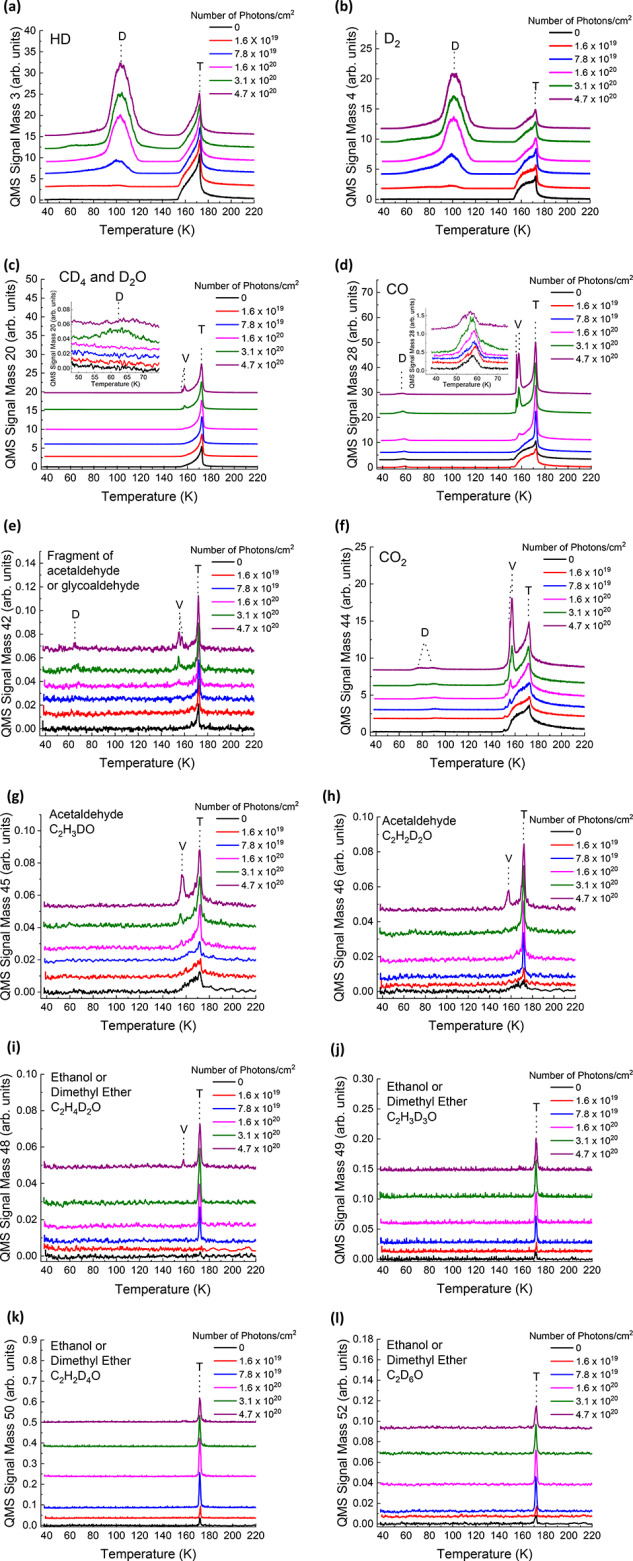
ΔP-TPD profiles for products resulting from 193 nm photon irradiation of 80 ML (H_2_O) ASW/10 ML CD_3_OD/10 ML (H_2_O) ASW/Ru(0001) sandwich films (38 K) at fluences from 0 to 4.7 × 10^20^ photons/cm^2^: (a) *m*/*z* = 3, (b) *m*/*z* = 4, (c) *m*/*z* = 20, (d) *m*/*z* = 28, (e) *m*/*z* = 42, (f) *m*/*z* = 44, (g) *m*/*z* = 45, (h) *m*/*z* = 46, (i) *m*/*z* = 48, (j) *m*/*z* = 49, (k) *m*/*z* = 50, and (l) *m*/*z* = 52. Note that the *Y*-axis corresponds to the actual (relative) intensity of each of the measured *m*/*z* values and that the ΔP-TPD profiles are offset for clarity. For each *m*/*z*, the different peaks are labeled as follows: “D” for the diffusion TPD peak, “V” for the volcano TPD peak, and “T” for the trapped TPD peak.

When sandwich films of 80 ML (H_2_O) ASW/10 ML CD_3_OD/10 ML (H_2_O) ASW/Ru(0001) are irradiated by 3.1 × 10^20^ photons/cm^2^ (193 nm excimer laser wavelength, 1.5 mJ/pulse energy at the sample), new products are formed. New peaks at *m*/*z* = 3, 4, 20, 28, 42, 44, 45, 46, 48, 49, 50, and 52 are detected by conducting 3D-ΔP-TPD (see Figure 1) measurements upon completion of the photon irradiation. In these measurements, masses in the range of 3−16 and 19−70 amu are scanned as the film is heated at a constant rate of 0.5 K/s. (These masses are assigned to the following products (as indicated in Figures 3a–l): *m*/*z* = 3 to HD, *m*/*z* = 4 to D_2_, *m*/*z* = 20 to CD_4_ or D_2_O, *m*/*z* = 28 to CO, *m*/*z* = 42 to a fragment of acetaldehyde or glycolaldehyde, *m*/*z* = 44 to CO_2_, *m*/*z* = 45–46 to acetaldehyde, and *m*/*z* = 48–52 to either ethanol or dimethyl ether. See the discussion below in Section 3.3 for further details on how the product assignments are made. Once the masses of the new products are identified, the effect of the number of photons (fluence) on the amount of products formed is explored. The same sandwich films (as mentioned above) are irradiated by photons varying in fluence from 1.6 × 10^19^ to 4.7 × 10^20^ photons/cm^2^. ΔP-TPD experiments tracking masses that were detected in the preceding 3D -ΔP-TPD experiments and masses corresponding to the intact CD_3_OD molecule and its main electron impact fragmentation pattern within the QMS ionizer (*m*/*z* = 32, 34, and 36) are conducted. The ΔP-TPD profiles show that the amounts of the products (calculated by taking the area under the ΔP-TPD peaks) increase as the fluence increases until a certain point when saturation has been reached (Figures 3a–l). The peaks of masses corresponding to intact CD_3_OD decrease as the fluence increases (not shown). The ΔP-TPD profiles display three types of peaks: a “diffusion” (low temperature) peak (D), a “volcano” peak (V), and a “trapped” peak (T). These peaks can provide information about the newly formed product’s chemical environment and location within the ASW film as well as its strength of interaction with the ASW host. This information is useful in determining the identity of a molecule observed at a given mass *m*/*z*. Products that interact weakly with ASW and have the capability to diffuse through ASW pores display a “diffusion” (D) peak.^46^ This corresponds to the diffusion of molecules through ASW pores located near the ASW/vacuum interface before any measurable structural changes occur in the ASW film. In addition, weakly interacting molecules that are located further from the ASW/vacuum interface will desorb explosively in a “volcano” (V) peak^47,48^ at the crystallization temperature of ASW. Molecules that interact strongly with water will desorb in the “trapped” (T) peak, together with the multilayer desorption of the water molecules from the substrate. Because the photochemical products each interact differently with the ASW host film, there is no expectation that all three peaks will be observed for each *m*/*z* measured in a ΔP-TPD experiment. Furthermore, the presence or lack of a specific type of peak will aid in identifying the photochemical products, as this can give information about the strength of interaction between the reaction product and the ASW host film.

**Figure 4:**
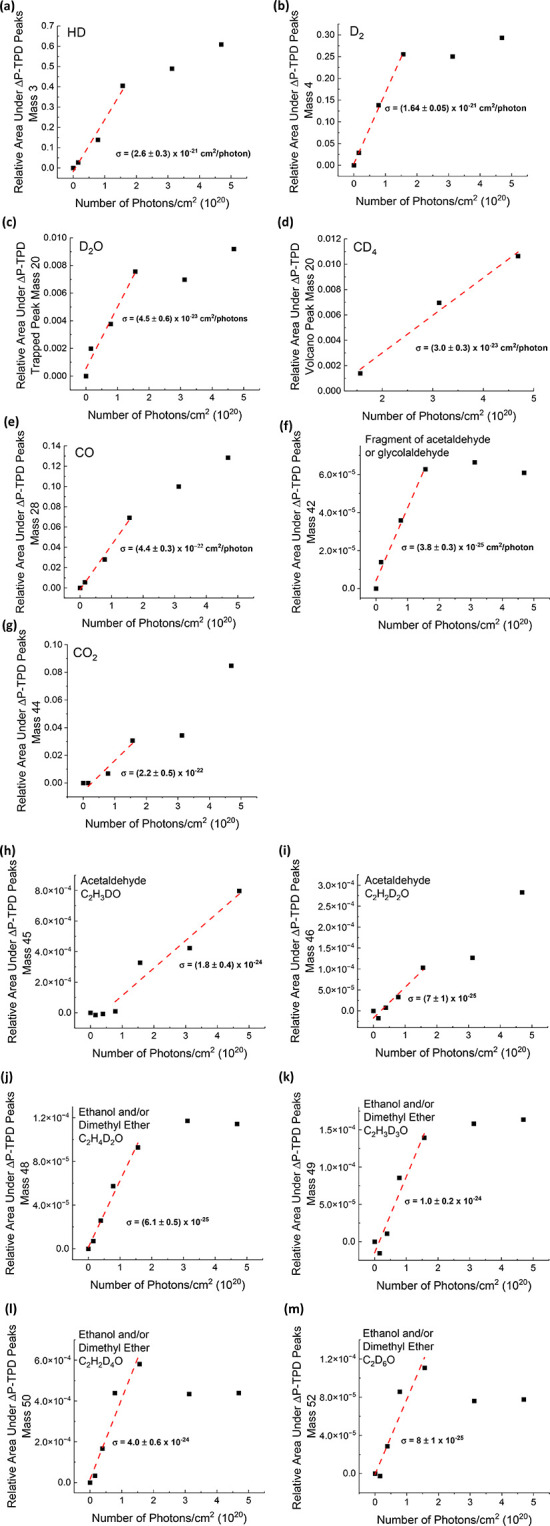
Reactivity cross sections (σ in cm^2^/photon units) for the products of 193 nm photon irradiation of 80 ML (H_2_O) ASW/10 ML CD_3_OD/10 ML (H_2_O) ASW/Ru(0001) sandwich films (38 K) at fluences from 0 to 4.7 × 10^20^ photons/cm^2^. The area under the ΔP-TPD peaks (presented in Figure 3) for a given *m*/*z* relative to the area under the ΔP-TPD peaks obtained from clean 10 ML CD_3_OD on Ru(0001) (including its fragments within the QMS) is plotted versus the photon fluence for the following masses: (a) *m*/*z* = 3, (b) *m*/*z* = 4, (c) *m*/*z* = 20 (D_2_O), (d) *m*/*z* = 20 (CD_4_), (e) *m*/*z* = 28, (f) *m*/*z* = 42, (g) *m*/*z* = 44, (h) *m*/*z* = 45, (i) *m*/*z* = 46, (j) *m*/*z* = 48, (k) *m*/*z* = 49, (l) *m*/*z* = 50, and (m) *m*/*z* = 52. The assigned molecular products are indicated for each *m*/*z* value.

Reactivity cross sections for the formation of products following photon irradiation (energy = 6.4 eV (193 nm), power = 1.5 mJ/pulse) are calculated in cm^2^/photon (Figures 4a–l). For each *m*/*z*, the areas under all observed ΔP-TPD peaks (in Figure 3) in the control experiment (no photons) were summed up and subtracted from the peak area for all observed peaks at each fluence. In this way, only the increase in area under the peaks that result from photon irradiation is considered when calculating the reactivity cross section for each product. The normalized area under the ΔP-TPD peaks for each *m*/*z* (relative to ΔP-TPD of clean 10 ML of CD_3_OD adsorbed on Ru(0001), including its fragments within the QMS) is plotted versus the fluence in photons/cm^2^. Calculating the slope of the initial growth rate of the products gives the cross section (σ) for the formation of each product in cm^2^/photon units. The largest reactivity cross sections were obtained for *m*/*z* = 3, 4, 28, and 44. Cross sections are summarized in Table 1.

**Table 1:** Reactivity Cross Sections (σ in Units of cm^2^/Photon) Are Presented for the Formation of Products in 80 ML (H_2_O) ASW/10 ML CD_3_OD/10 ML (H_2_O) ASW/Ru(0001) Sandwich Films upon Irradiation with 193 nm Photons

mass (amu)	cross section (cm^2^/photon)
3	(2.6 ± 0.3) × 10^–21^
4	(1.65 ± 0.05) × 10^–21^
20 (D_2_O)	(4.5 ± 0.6) × 10^–23^
20 (CD_4_)	(3.0 ± 0.3) × 10^–23^
28	(4.4 ± 0.3) × 10^–22^
42	(3.8 ± 0.3) × 10^–25^
44	(2.2 ± 0.5) × 10^–22^
45	(1.8 ± 0.4) × 10^–24^
46	(7 ± 1) × 10^–25^
48	(6.1 ± 0.5) × 10^–25^
49	(1.0 ± 0.2) × 10^–24^
50	(4.0 ± 0.6) × 10^–24^
52	(8 ± 1) × 10^–25^

### 3.3 Photoproduct Identification

The products of 193 nm photon irradiation of 80 ML (H_2_O) ASW/10 ML CD_3_OD/10 ML (H_2_O) ASW/Ru(0001) sandwich films at 38 K are identified based on their *m*/*z*, their nature of interaction with the ASW film (whether the “diffusion” (D), “volcano” (V), or “trapped” (T) TPD peaks increase in intensity as a result of increasing photon fluence), and support from the literature. Masses *m*/*z* = 3 and 4 are assigned to the formation of HD and D_2_, respectively (Figures 3a,b). Both ΔP-TPD profiles display large “diffusion” peaks at 100 K that increase with increasing fluence. The amounts of *m*/*z* = 3 and 4 in the “trapped” peaks decrease with increasing photon fluence, indicating that the ΔP-TPD signal is likely originating from electron impact fragmentation patterns of remaining water and methanol within the QMS ionizer. Additionally, control measurements of nonirradiated CD_3_OD@ASW films display the “trapped” peak for *m*/*z* = 3 and 4 (not shown), which is a further evidence that this peak originates from fragmentation of the methanol parent molecule within the QMS ionizer. No “volcano” peak is observed for these masses. The “hump” that is observed in the ΔP-TPD profiles around the temperature of a typical “volcano” peak (even for films that are not irradiated with photons) is attributed to the desorption of methanol or methanol–water hydrogen bonded complexes. The “hump” decreases in intensity as the photon fluence increases, indicating that less intact methanol remains in the ASW film. The newly formed HD and D_2_ mostly diffuse through the ASW pores to desorb to the vacuum. The reactivity cross section for the formation of HD at *m*/*z* = 3 (σ = (2.6 ± 0.3) × 10^–21^) is larger than for the formation of D_2_ at *m*/*z* = 4 (σ = (1.64 ± 0.05) × 10^–21^) (Figures 4a,b). Irradiation of ASW by 193 nm photons can apparently lead to the dissociation of water molecules in the film.^27−30,49^ Because there is significantly more H_2_O than CD_3_OD in the sandwich structure, and therefore more H atoms are produced through photon irradiation, it is reasonable that HD is formed at a higher probability than D_2_. Hydrogen molecules (*m*/*z* = 2), which are also expected to be formed, were not monitored due to the large background of this mass in the UHV chamber.

Mass *m*/*z* = 20 is assigned to both deuterated water (D_2_O) and deuterated methane (CD_4_) (Figure 3c). In the literature, both of these products are proposed to form as a result of the direct photodissociation of methanol.^33^ The ΔP-TPD profile for *m*/*z* = 20 displays four peaks that increase with increasing photon irradiation: a “diffusion” peak, *two* “volcano” peaks (separated by 1–2 degrees), and a “trapped” peak. The two “volcano” peaks are probably observed because of homogeneous nucleation at slightly different crystallization temperatures of areas containing H_2_O–CD_3_OD as the dominant complex in addition to areas of pure H_2_O. Because CD_4_ is hydrophobic and rather inert, the molecules of *m*/*z* 20 that desorb in the “diffusion” and “volcano” peaks are attributed exclusively to the formation of CD_4_ molecules. The newly formed D_2_O molecules desorb with the water (H_2_O) multilayer and therefore are exclusively contained in the “trapped” peak at 172 K. The “diffusion” and “volcano” peaks for *m*/*z* = 20 are only observed when the photon irradiation is ≥3.1 × 10^20^ cm^2^/photon. At this fluence, the relative area under the “trapped” peak for *m*/*z* = 20 reaches saturation. The cross section of formation of D_2_O ((4.5 ± 0.6) × 10^–23^ cm^2^/photon) is 50% larger than that of CD_4_ ((3.0 ± 0.3) × 10^–23^ cm^2^/photon) (see Figures 4c,d). This is a reasonable result, having only half of the D atoms (in D_2_O) that are obtained from dissociating the parent CD_3_OD molecule.

The ΔP-TPD profile for *m*/*z* = 28 reveals “diffusion”, “volcano”, and “trapped” peaks (Figure 3d). This profile is similar to what has been observed in the literature for the desorption of CO from ASW films,^50^ and therefore *m*/*z* = 28 is assigned to CO. At low fluences (<1.6 × 10^20^ photons/cm^2^) the “trapped” peak increases in intensity with the number of photons while the “diffusion” and “volcano” peaks show minimal growth. When the fluence is increased above 1.6 × 10^20^ photons/cm^2^, the “diffusion” and “volcano” peaks increase more noticeably. Because water can form a relatively weak hydrogen bond with CO,^51^ those CO molecules which form hydrogen bonds with surrounding water molecules will desorb with the water multilayer (“trapped” peak) as opposed to CO molecules in the “volcano” peak. As the amount of CO produced increases, more CO desorbs in the “diffusion” and “volcano” peaks. The reactivity cross section for the formation of CO is (4.4 ± 0.3) × 10^–22^ cm^2^/photon (Figure 4e), an order of magnitude larger than that of CD_4_ or D_2_O. The “volcano” desorption of CO also displays a “double peak” separated by 1–2 degrees, indicating a nonhomogeneous crystallization throughout the ASW-CD_3_OD film (see Figure 3d).

The ΔP-TPD profile for mass *m*/*z* = 42 (Figure 3e) displays one to three peaks, depending on the number of photons. At low fluences (≤7.8 × 10^19^ photons/cm^2^), only the “trapped” peak is observed. When the fluence in increased, the two “volcano” peaks are apparent. While we cannot completely rule out the presence of a “diffusion” peak at high fluences, the signal is below our detection limit and therefore excluded from our cross-section calculations. The preference for *m*/*z* = 42 desorbing in the “trapped” peak indicates that this molecule interacts relatively strongly with the water molecules in the ASW film. We could not find a stable molecule of *m*/*z* = 42 that could form in the current photochemical setup. Therefore, the signal at *m*/*z* = 42 is likely due to fragmentation within the QMS ionizer of a larger molecule, which was formed through photon irradiation of the film. Mass *m*/*z* = 42 could be a fragment of acetaldehyde or glycolaldehyde, which are both possible products of photon irradiation of methanol^31,52^ and can both form hydrogen bonds with water. The cross section of formation for mass *m*/*z* = 42 is (3.8 ± 0.3) × 10^–25^ cm^2^/photon (Figure 4f).

Mass *m*/*z* = 44 is assigned to formation of CO_2_, which is documented as a product of UV photon irradiation of methanol.^31^ The ΔP-TPD profile displays three peaks, all increase in intensity with the photon fluence (Figure 3f). The relative area under the ΔP-TPD peaks does not reach saturation for the fluences tested here. The reactivity cross section of formation of CO_2_ is (2.2 ± 0.5) × 10^–22^ cm^2^/photon (Figure 4g), expected to be similar to, but slightly smaller than, the cross section for CO formation.

The masses *m*/*z* = 45–52 can be assigned to acetaldehyde, ethanol, or dimethyl ether of varying H/D ratios. The differences in the ΔP-TPD profiles for these masses indicate that more than one product is observed in this mass range. Of the three molecules mentioned above, *m*/*z* = 45 can only correspond to acetaldehyde. Because the ΔP-TPD profiles for masses *m*/*z* = 45 and 46 are similar to one another (Figures 3g,h), they probably both represent acetaldehyde with different H/D ratios. Mass *m*/*z* = 45 corresponds to acetaldehyde containing one deuterium atom, while mass 46 corresponds to acetaldehyde containing two deuterium atoms. Both *m*/*z* = 45 and 46 ΔP-TPD profiles display increasing “volcano” and “trapped” peaks at high photon fluences. The relative areas under the ΔP-TPD peaks for these masses do not reach saturation at the maximum fluence tested here. Although most of the molecules desorb in the “trapped” peak, the eventual appearance of the “volcano” TPD peak at higher laser fluence indicates that the molecule’s interaction with water is not too strong. This fits with the assignment to acetaldehyde, which can form one hydrogen bond with water, but still prefers to form clusters at the interface when deposited on water–ice surface.^53^ Some hydrogen bonds are formed between water and acetaldehyde resulting in the codesorption of the two molecules. Acetaldehyde molecules that do not form hydrogen bonds with water due to unfavorable relative orientation are ejected in the “volcano” peak.

**Figure 5:**
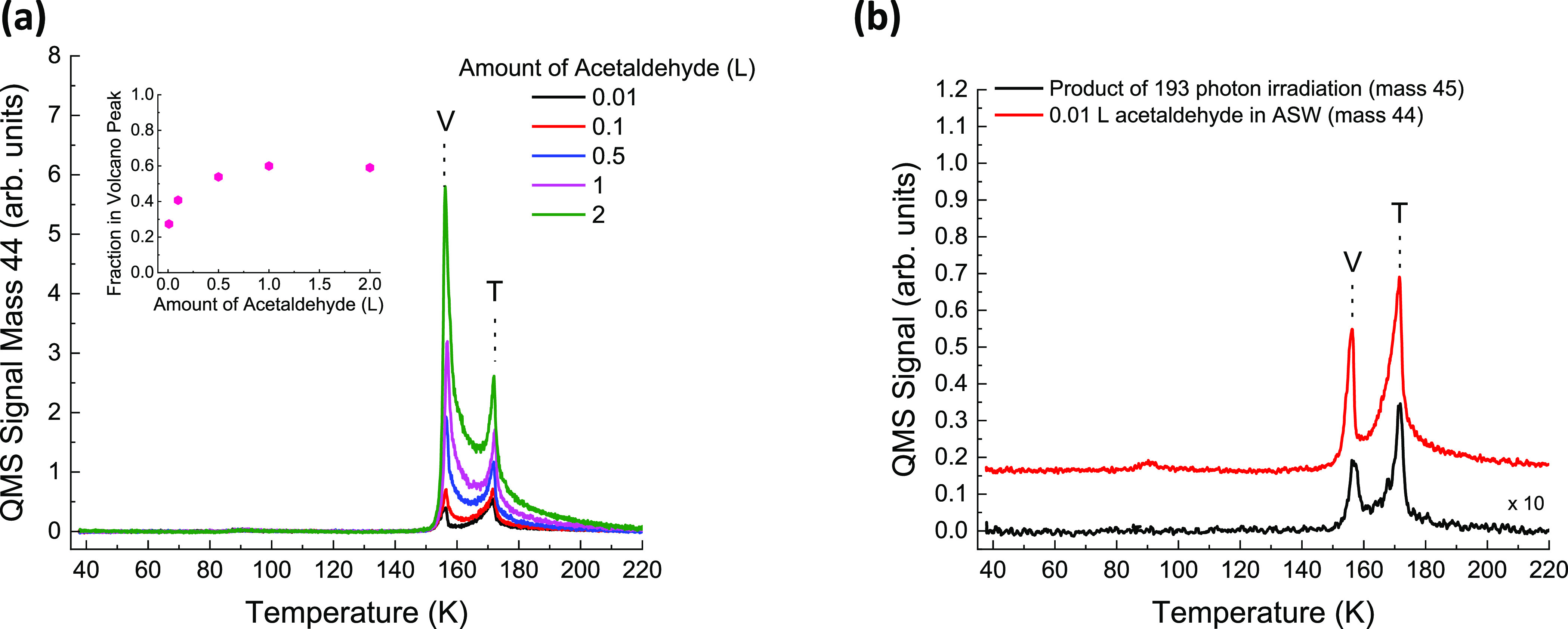
Comparison of ΔP-TPD profiles of acetaldehyde in ASW to observed photoproducts of mass 45 and 46. In (a) the initial amount of acetaldehyde is varied from 0.01 to 2 L in a sandwich structure 80 ML (H_2_O) ASW/*X* L acetaldehyde/10 ML (H_2_O) ASW/Ru(0001) without any photon irradiation. The inset shows the fraction of acetaldehyde (at mass 44) that desorbs in the volcano peak. (b) ΔP-TPD profile for 0.01 L acetaldehyde in ASW (no UV irradiation) is compared to that of photoproduct at mass *m*/*z* = 45 when a 80 ML (H_2_O) ASW/10 ML CD_3_OD/10 ML (H_2_O) ASW/Ru(0001) film at 38 K is irradiated by 193 nm photons at a fluence of 4.7 × 10^20^ photons/cm^2^. The profile for 0.01 L acetaldehyde in ASW is offset for clarity. The peaks are labeled either “V” for volcano or “T” for trapped TPD peaks.

To confirm the assignment of masses 45 and 46 to acetaldehyde, the ΔP-TPD profiles of nonirradiated acetaldehyde molecules sandwich structures of (90 – *X*) ML (H_2_O) ASW/*X* L acetaldehyde/10 ML (H_2_O) ASW/Ru(0001) are studied, tracking *m*/*z* = 44 which is the mass of the parent molecule (nondeuterated) acetaldehyde. The acetaldehyde amount is varied from 0.01 to 2 L (Figures 5a,b). The ΔP-TPD profiles show that as the amount of acetaldehyde in the sandwich decreases, the fraction of molecules desorbing in the volcano peak decreases (inset in Figure 5a). In the current setup, doses below 0.01 L cannot be accurately deposited. The ΔP-TPD profile of the smallest dose of acetaldehyde (0.01 L) is similar in shape (although higher in intensity) to that obtained for mass *m*/*z* = 45 as a result of 193 nm photon irradiation at a fluence of 4.7 × 10^20^ photons/cm^2^ (Figure 5b). This comparison indicates the high probability that masses *m*/*z* = 45 and 46 do in fact correspond to the formation of acetaldehyde. The cross sections of formation of *m*/*z* = 45 and 46 are (1.8 ± 0.4) × 10^–24^ and (7 ± 1) × 10^–25^ cm^2^/photon, respectively (Figures 4h,i).

Masses 48, 49, 50, and 52 can be assigned to either ethanol or dimethyl ether. Dimethyl ether can form hydrogen bonds with water in an analogous structure to the water–water hydrogen bond. This hydrogen bond is slightly stronger than the water dimer hydrogen bond.^54−56^ Ethanol also forms stronger hydrogen bonds with water than water does with itself.^57^ Without further spectroscopy we cannot confirm the identity of this product. Because both dimethyl ether and ethanol form strong hydrogen bonds with water, and only display one peak (“trapped”) in the ΔP-TPD spectra (Figures 3i–l), these molecules are both plausible products. The mass range represents varying isotopes of these products, with mass *m*/*z* = 52 representing the fully deuterated form of ethanol or dimethyl ether. The cross sections of formation for these products range from (6.1 ± 0.5) × 10^–25^ for mass 48 to (4.0 ± 0.6) × 10^–24^ for *m*/*z* = 50 (Figures 4j–m). The cross sections indicate that the most abundant isotope is that of *m*/*z* = 50, which represents a mostly deuterated molecule, with four deuterium atoms and only two hydrogen atoms. The least abundant isotope is one with four hydrogen atoms and two deuterium atoms (*m*/*z* = 48). The exact position of the D (or H) atoms in these molecules cannot be determined using our current experimental tools.

### 3.4 Mechanism of Product Formation

The products of 193 nm photon irradiation of the CD_3_OD@(H_2_O) ASW films can be formed through either direct photodissociation of the water and methanol molecules and/or through low-energy dissociative electron attachment (DEA). Previous research has shown that the irradiation of gaseous methanol with 193 nm photons (6.4 eV) leads to the photodissociation of methanol.^58^ In the amorphous solid phase, the photodissociation of methanol at 157 nm (7.9 eV) has been studied,^37,59^ while such studies at 193 nm have not been reported in the literature so far. For understanding the mechanism of the photodissociation observed here, we rely on some mechanisms proposed for the gas phase dissociation of methanol at 193 nm.

Several dissociation pathways have been proposed for the absorption of 193 nm light by methanol in the gas phase.^33,58^ Additionally, because the photon energy (6.4 eV) is larger than the work function of the clean Ru(0001) substrate (Φ = 5.52 eV^60^), photoelectrons are emitted from the substrate when the CD_3_OD@(H_2_O) ASW films are irradiated by the 193 nm photons. Additionally, the adsorption of H_2_O and CD_3_OD on Ru(0001) lowers the work function even further at the layer thickness of 100 ML described here, as observed in measurements performed using a Kelvin probe (not shown here). Therefore, these photoelectrons probably have an energy of 2–3 eV and can attach themselves to CD_3_OD or H_2_O molecules in the sandwich films through a DEA mechanism. This leads to the formation of a transient, energetic anion which then fragments into an anion and neutral species (e.g., CD_3_O^–^ + D·).^61,62^ These can subsequently act as reactants that lead to the formation of new products that differ from the products of direct photodissociation of methanol and water that lead primarily to neutral radicals.

**Figure 6:**
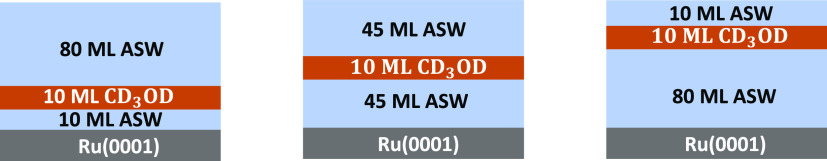
Schematic of position of 10 ML CD_3_OD within ASW with a total film thickness of 100 ML.

To determine whether the products observed in this study are being formed through photodissociation or DEA, CD_3_OD@(H_2_O) ASW sandwich films varying in the initial position of the CD_3_OD layers relative to the substrate are prepared at 38 K (Figure 6). Because the CD_3_OD is not dispersed throughout the ASW at 38 K, but rather forms a distinct layer within the ASW film,^45^ the distance between the CD_3_OD layers and the Ru(0001) substrate can be controlled. In this way, the effect of the photoelectrons on the product formation can be evaluated. Products that are not affected by the distance between the Ru(0001) and the CD_3_OD layers are most likely formed through direct photodissociation of the CD_3_OD molecules. Products that are affected by the initial CD_3_OD position are primarily formed through DEA. Products formed through DEA will show a decrease in the amount of products produced (seen as a decrease in the area under the ΔP-TPD profile for a given *m*/*z*) when the CD_3_OD is initially placed further from the Ru(0001) substrate. This is because not all the photoelectrons will be able to travel as far in the film (from the metal substrate up toward the ASW–vacuum interface) and will also lose energy before arriving at the CD_3_OD layers, leading to a decrease in the amount of products observed.

In this study, masses *m*/*z* = 20, 28, 42, 44, 46, and 48 were barely affected by the change in initial CD_3_OD distance from the Ru(0001) substrate. This indicates that these products are formed through direct photodissociation of methanol and water molecules by the 193 nm photons. Interestingly, the ΔP-TPD signal for *m*/*z* = 45 (assigned to acetaldehyde) increases when initially placed further from the Ru(0001) (relative area under the peak increases by 30% when the CD_3_OD is initially placed near the top of the film as opposed to near the Ru(0001) substrate). This could indicate that the emitted photoelectrons are leading to the dissociation of this product (*m*/*z* = 45) that is also formed through photodissociation. This is more likely to occur when the CD_3_OD is initially placed closer to the Ru(0001) substrate. In other words, the competing DEA mechanism that leads to further decomposition of the acetaldehyde product while the 193 nm photons strike the film is most effective when the alcohol layer is closest to the ruthenium substrate. In contrast, for *m*/*z* = 3, 4, 49, 50, and 52 the ΔP-TPD signals decrease as the CD_3_OD is initially placed further from the Ru(0001) substrate. This indicates that these products are formed primarily through DEA, a mechanism that leads to the highest yield when the CD_3_OD layers were initially placed closest to the substrate. DEA appears to be the dominant mechanism for the formation of products in this system. The most abundant products are HD, D_2_, CO, and CO_2_ with relative abundances to CD_3_OD of 50%, 25%, 10%, and 3.4%, respectively. HD and D_2_ are formed through DEA, while CO and CO_2_ are formed through direct photodissociation of methanol. To form HD, a hydrogen atom from water reacts with a deuterium atom from CD_3_OD in the following proposed mechanism:Formation of H atom via DEA of H_2_O:
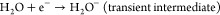

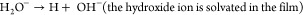
Formation of D atom via DEA of CD_3_OD:
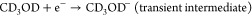

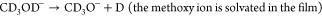
Finally, the hydrogen atom and deuterium atom diffuse in the film and combine to form HD:
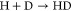
Molecular deuterium (*m*/*z* = 4) forms in a similar fashion, but from two deuterium atoms resulting from DEA of CD_3_OD:
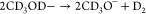
Both CO (*m*/*z* = 28) and CO_2_ (*m*/*z* = 44) are formed through direct photodissociation of the methanol, in the following mechanism proposed by Öberg et al.:^31^
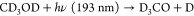

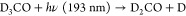

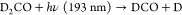

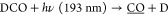

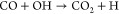


Interestingly we observe very little formaldehyde (*m*/*z* = 30–32) which is often reported as a major product of photodissociation^31,33,63^ and electron-induced processing^64^ of methanol. We also observe relatively large amounts of CO, CO_2_, HD, and D_2_. This could indicate that larger products (such as formaldehyde, ethanol, dimethyl ether, acetaldehyde, and glycolaldehyde) that initially form upon irradiation by 193 nm photons could eventually be further dissociated by incoming photons or photoelectrons. Although products are not detected below photon irradiations of 1.6 × 10^19^ photons/cm^2^, the fluence used in this study may have been too large to observe those relatively large molecular products in high yields. In addition, the initial layer of methanol described in this study is thicker (contains more methanol molecules) than previous studies of pure layers of methanol or methanol embedded in ASW. This difference in methanol density probably shifts the product distribution to what is reported here as opposed to what is reported in previous studies, where the layers of methanol are at smaller densities.

### 3.5 Undetectable Electric Field Effect on the Photochemistry of CD_3_OD@(H_2_O) ASW Sandwich Films

We are interested in understanding how strong electric fields may affect chemical reactions. Here, we have a model system for studying the photochemistry of CD_3_OD@ASW under the influence of an electric field. By charging the CD_3_OD@ASW films with low-energy Ne^+^ ions, a stable charge layer of H_3_O^+^ ions is generated. This is accomplished through a charge transfer mechanism in which the Ne^+^ ions take electrons from surface water molecules in the ASW film, resulting in the formation of neutral Ne atoms that scatter back to the vacuum and positive water cations (H_3_O^+^) that remain on the ASW–vacuum interface. This way, one can form a nanocapacitor with a maximum field strength of 2 × 10^8^ V/m.^44^ Because the largest amount of products is observed when the CD_3_OD is initially placed closer to the substrate [80 ML (H_2_O) ASW/10 ML CD_3_OD/10 ML (H_2_O) ASW/Ru(0001)], the effect of the electric field was studied for this film.

When these films are charged following Ne^+^ ion collisions at 85 eV for 10 min (to obtain the maximum electric field strength) prior to 193 nm photon irradiation, the electric field is practically neutralized by the photoelectrons emitted from the Ru(0001). Possible reactions of the negative ions discussed above (^−^OH and CD_3_O^–^) with the H_3_O^+^ generated by the Ne^+^ ions may also lead to neutralization of the electric field. This is observed by monitoring change in contact potential difference (ΔCPD) as a result of the charging and photon irradiation processes. When the films are first irradiated with 193 nm photons and subsequently charged with 85 eV Ne^+^ ions for 10 min, the film remains positively charged and the electric field strength remains at 2 × 10^8^ V/m. The electric field is calculated based on the plate capacitor equation described by^44^
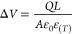
where Δ*V* is the measured ΔCPD obtained as a result of the positive charging described above, *Q* is the accumulated positive charge, *L* is the layer thickness of the CD_3_OD@ASW film, *A* is the area of the film exposed to the impinging charges, ε_0_ is the vacuum permittivity, and ε_(*T*)_ is the static dielectric constant of the material between the plates. Here we assume that ε_(*T*)_ of pure ASW at 38 K (ε_(*T*)_ = 3.2) and that for the methanol–water sandwich film structure at the same temperature are identical. The film thickness (*L*) is calculated by converting the monolayers of water and methanol to nanometers and the voltage developed across the film is experimentally measured in situ. The electric field strength is calculated by the following equation:
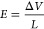
Because in the current setup the electric field is generated after the photon irradiation is complete, the electric field cannot influence the initial photodissociation event, but it has the potential to affect the reactivity of the fragments formed through 193 nm photon irradiation due to their limited mobility at 38 K and during sample heating in the ΔP-TPD measurements.

**Figure 7:**
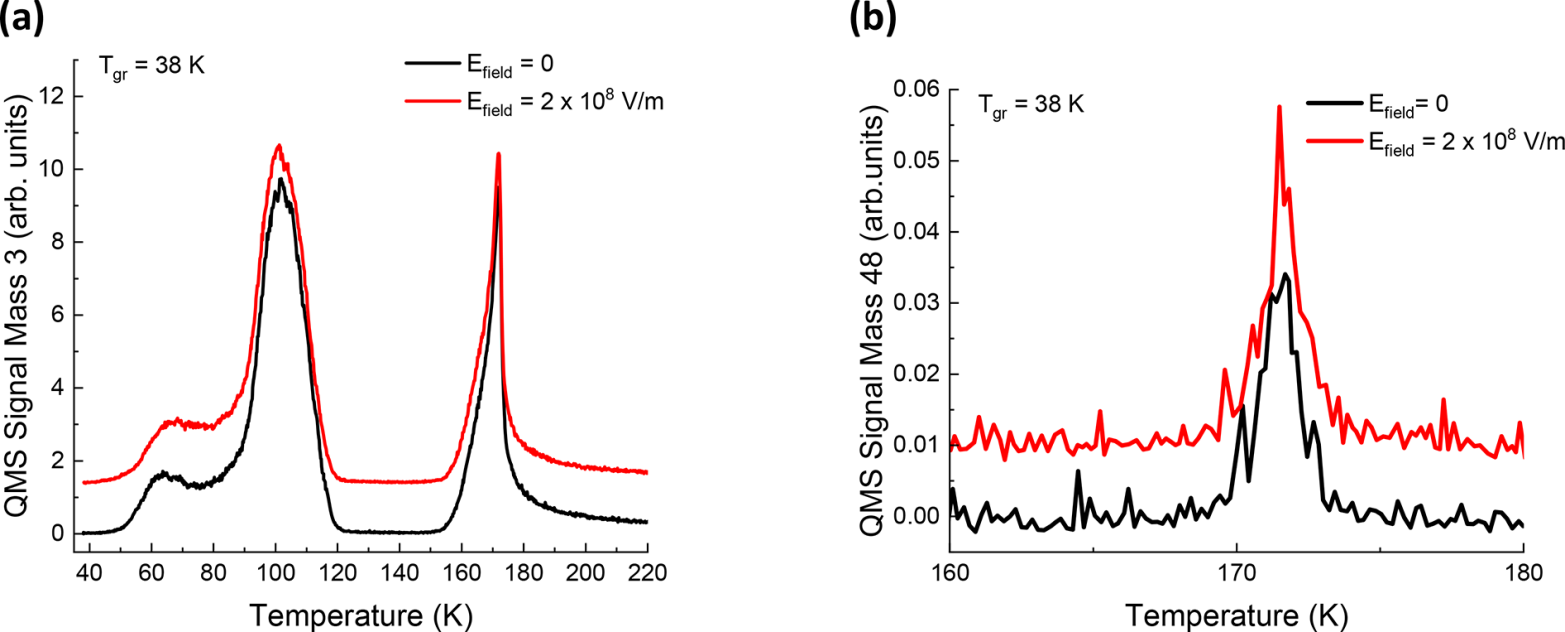
ΔP-TPD profiles showing the (no) effect of an electric field on the formation of (a) *m*/*z* = 3 (HD) and (b) *m*/*z* = 48 (ethanol or dimethyl ether) when films are grown and irradiated at 38 K in the presence of an electric field. The Δ*P*-TPD profiles are offset for clarity.

Films of 80 ML (H_2_O) ASW/10 ML CD_3_OD/10 ML (H_2_O) ASW/Ru(0001) deposited at 38 K are irradiated with 3.1 × 10^20^ photons/cm^2^ (193 nm, 6.4 eV, 1.5 mJ/pulse at the sample) and subsequently charged by low-energy Ne^+^ ions (85 eV) for 10 min to obtain maximum charging and electric field strength of 2 × 10^8^ V/m. Subsequently, a ΔP-TPD experiment is performed, tracking the masses *m*/*z* that were observed in the photochemical experiments described in Sections 3.2 and 3.3. The ΔP-TPD profiles of each mass (*m*/*z*) obtained with and without the electric field are compared to evaluate the potential effect of the electric field on the product formation. Our conclusion so far is that under the described conditions no measurable effect of the electric field was observed (Figures 7a,b). The positive charges are discharged from the CD_3_OD@ASW film at a peak temperature of 58 K, when the film is grown at 38 K. This means that the electric field is not present at higher temperatures and can only influence the reaction pathway of energetic reactants in a small temperature range, where the molecules are still relatively immobile. This may not be a high enough temperature range for the influence of the electric field to be observed.

**Figure 8:**
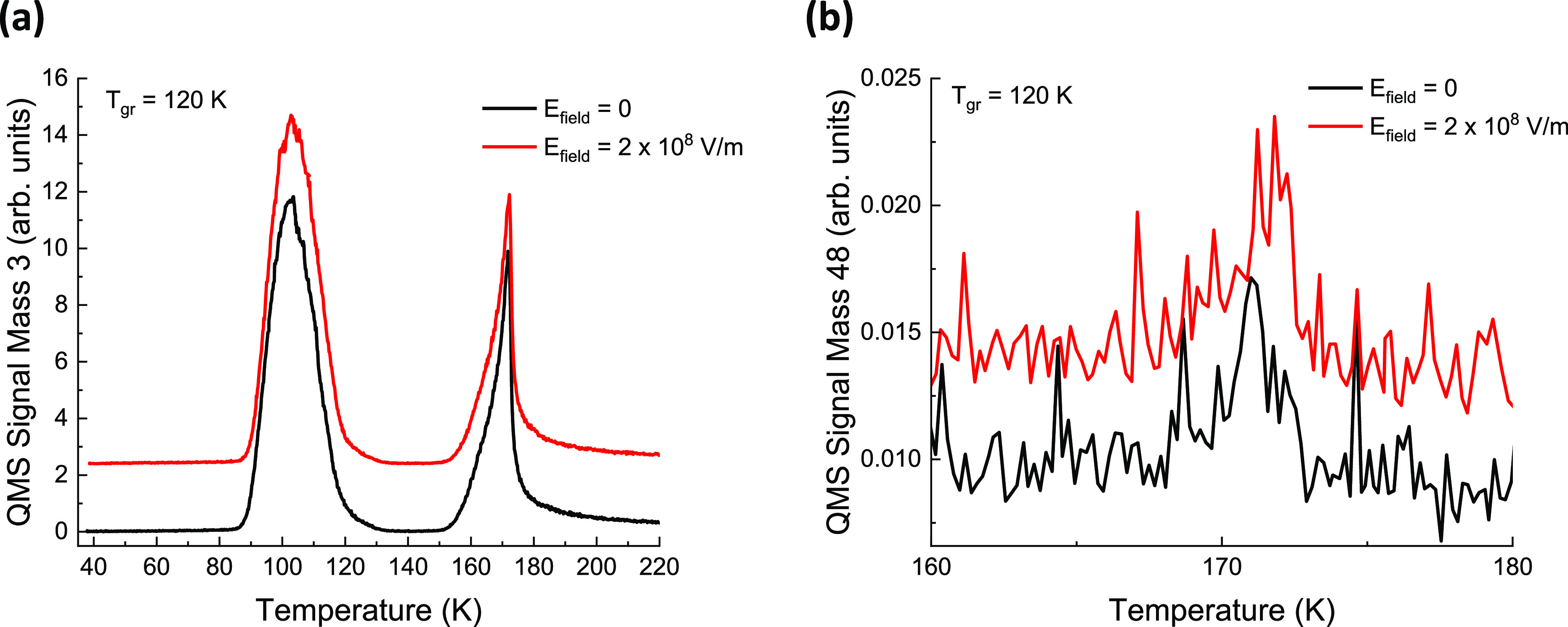
ΔP-TPD profiles showing the (no) effect of an electric field on the formation of (a) *m*/*z* = 3 (HD) and (b) *m*/*z* = 48 (ethanol or dimethyl ether) when films are grown at 120 K and irradiated at 38 K. The ΔP-TPD profiles are offset for clarity.

To expand the temperature range under the influence of the electric field, films of 80 ML (H_2_O) ASW/10 ML CD_3_OD/10 ML (H_2_O) ASW/Ru(0001) are deposited (grown) at 120 K. The films are then cooled to 38 K and irradiated with 3.1 × 10^20^ photons/cm^2^ followed by charging with 85 eV Ne^+^ ions to a maximum electric field strength of 2 × 10^8^ V/m. Because the morphology and porosity of ASW films are affected by the growth temperature,^10,65−70^ the photochemistry may also be affected by a change in growth temperature. When the films are grown at 120 K, the ASW films are more compact, and the CD_3_OD molecules are almost homogeneously dispersed throughout the ASW film^45^ because of higher mobility of the molecules at this temperature. This could lead to a slight decrease in the amount of products formed upon irradiation with 193 nm photons. Additionally, the peak discharge temperature of the positive charges shifts (coupled to the growth temperature) all the way to 118 K,^44^ giving the reactants produced by the photon irradiation both thermal energy and time to recombine under the influence of the electric field. Nevertheless, also under these conditions, no measurable effect of the electric field was observed (Figures 8a,b).

In previous reports, it was theoretically predicted that a field strength on the order of 10^9^ V/m is required to observe effects on chemical reactions.^71^ With water as the dielectric material and our current experimental setup, field strengths of this kind cannot be achieved. Additionally, it is possible that the water and methanol molecules slightly rotate to align themselves with the electric field, thereby screening and diminishing the actual electric field strength within the film. It is, however, interesting to note that more subtle molecular motions and vibrational Stark effect were reported at similar electric field strengths generated within ASW films.^38−41^

## 4 Conclusions

Photochemical products are observed as the result of 193 nm photon irradiation of CD_3_OD@ASW sandwich films grown and irradiated at 38 K. Products are formed through DEA in addition to direct photodissociation of methanol and water molecules. The products are most abundant when the CD_3_OD is initially placed closer to the Ru(0001) substrate in a 80 ML (H_2_O) ASW/10 ML CD_3_OD/10 ML (H_2_O) ASW/Ru(0001) sandwich structure. This is because DEA processes are more likely to occur when the CD_3_OD is initially placed closer to the Ru(0001) substrate. However, at the same time, further decomposition of radical products is also more probable (as in the case of acetaldehyde). The main photochemical products observed in this study are HD, D_2_, CO, and CO_2_. Varying the initial position of the 10 ML layer of CD_3_OD indicates that the HD and D_2_ are formed primarily through DEA of H_2_O and CD_3_OD molecules by photoelectrons, while CO and CO_2_ are primarily formed through direct photodissociation of the CD_3_OD molecules. Other products, such as D_2_O, CD_4_, acetaldehyde, and ethanol or dimethyl ether, are formed in smaller probability. Reactivity cross sections of formation were calculated for the observed products from the slope of the initial growth of product quantity vs number of photons and range from (2.6 ± 0.3) × 10^–21^ cm^2^/photon for HD (*m*/*z* = 3) to (3.8 ± 0.3) × 10^–25^ cm^2^/photon for *m*/*z* = 42 (fragment of acetaldehyde or glycolaldehyde). When strong electric fields (up to 2 × 10^8^ V/m) are generated within the CD_3_OD@ASW sandwich films, no influence of the electric field on the product formation was observed. It is possible that this is due to the fact that the main products (HD, D_2_, CO, and CO_2_) are likely formed immediately following irradiation at 38 K and therefore cannot be affected by the electric field that is generated at a later stage. In addition, as theory suggested, stronger fields are necessary for such an effect to be measurable. However, these field strengths cannot be supported by ASW films as studied here.
